# The polyamine spermine induces the unfolded protein response via the MAPK cascade in *Arabidopsis*

**DOI:** 10.3389/fpls.2015.00687

**Published:** 2015-09-10

**Authors:** G. H. M. Sagor, Pratima Chawla, Dong W. Kim, Thomas Berberich, Seiji Kojima, Masaru Niitsu, Tomonobu Kusano

**Affiliations:** ^1^Graduate School of Life Sciences, Tohoku UniversitySendai, Japan; ^2^Biodiversity and Climate Research Center, Laboratory CenterFrankfurt am Main, Germany; ^3^Frontier Research Institute for Interdisciplinary Sciences, Tohoku UniversitySendai, Japan; ^4^Faculty of Pharmaceutical Sciences, Josai UniversitySakado, Japan

**Keywords:** calcium, mitogen activated protein kinase cascade, polyamine, spermine, unfolded protein response, UPR inducer

## Abstract

In *Arabidopsis* three basic region leucine zipper (bZIP) transcription factor genes, *bZIP17*, *bZIP28*, and *bZIP60*, play crucial roles in the unfolded protein response (UPR). Previously we found that *bZIP60* is one of the spermine-induced genes. Consequently we further investigated the response of all the three bZIP genes to spermine. Expression of *bZIP17*, *bZIP28*, and *bZIP60*, and also their target genes was activated by spermine application as well as in plants with elevated endogenous spermine levels. Furthermore, spermine activated the splicing of the *bZIP60* transcript mediated by the ribonuclease activity of inositol-requiring enzyme 1 and also recruited bZIP17 and bZIP60 proteins from endoplasmic reticulum to nucleus. We therefore propose that spermine is a novel UPR inducer. Moreover, induction of UPR by spermine required calcium-influx to the cytoplasm and the genes for mitogen-activated protein kinase kinase 9 (*MKK9*), mitogen-activated protein kinase 3 (*MPK3*) and *MPK6*. The result indicates that spermine-induced UPR is mediated by the MKK9-MPK3/MPK6 cascade in *Arabidopsis*.

## Introduction

Polyamines (PAs) are low-molecular mass, aliphatic compounds and they are ubiquitously present in all living organisms (Tabor and Tabor, 1984; Cohen, 1998). In plants, major PAs are putrescine (Put), spermidine (Spd), spermine (Spm), and thermospermine (T-Spm; Knott et al., 2007; Takano et al., 2012). PAs have important roles in various physiological processes not only in embryogenesis, growth, development and aging/senescence but also in adaptation responses to abiotic and biotic stresses (Kusano et al., 2008; Alcázar et al., 2010; Takahashi and Kakehi, 2010; Tiburcio et al., 2014; Berberich et al., 2015).

Polyamine precursors are ornithine and/or arginine. Ornithine is converted to Put by ornithine decarboxylase (ODC), while arginine is converted to Put via agmatine by three steps of enzyme reactions; arginine decarboxylase (ADC), agmatine iminohydrolase, and *N*-carbamoylputrescine amidohydrolase. Put is converted to Spd by Spd synthase, in which another substrate, decarboxylated *S*-adenosylmethionine (dcSAM), is required. dcSAM is synthesized by *S*-adenosylmethionine decarboxylase (SAMDC) from *S*-adenosylmethionine (SAM). Spd is further converted either to Spm by Spm synthase (SPMS) or to T-Spm by T-Spm synthase, also called ACAULIS5 (ACL5). SPMS and ACL5 also require dcSAM as another substrate (Kusano et al., 2008; Alcázar et al., 2010; Takahashi and Kakehi, 2010). In *Arabidopsis*, the *ODC* gene is absent (Hanfrey et al., 2001). Thus Put is synthesized only by the ADC pathway in this organism. On the other hand, PA catabolism is governed by two enzymes: the one is copper-dependent amine oxidase (CuAO) and the other is polyamine oxidase (PAO; Kusano et al., 2015). In *Arabidopsis*, there are more than 10 CuAOs and 5 PAOs. All the latter enzymes are characterized (Kusano et al., 2015).

As mentioned, involvement of PAs and their metabolism in the defense responses against pathogens is described (Walters, 2003a,b). PAs and their oxidative products by CuAO and PAO play crucial roles in plant defense responses (Berberich et al., 2015, and the references therein). When *Nicotiana tabacum* carrying the resistance gene *N* was infected by Tobacco mosaic virus (TMV), host plant displayed a hypersensitive response (HR). Once HR occurs, it is known that the tissues surrounding the TMV infection sites were killed by a suicidal reaction to prevent the virus multiplication. Yamakawa et al. (1998) reported that Spm enriched in the apoplastic space during HR triggered by *N. tabacum*-TMV pathosystem. Subsequently we found that exogenously applied Spm stimulates the phosphorylation activities of two MAPKs, wound-induced protein kinase (WIPK, Seo et al., 1995) and salicylic acid-induced protein kinase (SIPK, Zhang and Klessig, 1997), which trigger the expression of downstream defense genes in *N. tabacum* (Takahashi et al., 2003, 2004), and proposed it to call ‘Spm-signaling pathway.’ Massive identification of the Spm-responsive genes using a super serial analysis of gene expression (SuperSAGE) approach was performed in *Arabidopsis thaliana* (Mitsuya et al., 2009). The identified Spm-responsive genes behaved similarly during cucumber mosaic virus (CMV)-induced HR in *A. thaliana* (Takahashi et al., 1994; Mitsuya et al., 2009). One of them was a gene (*bZIP60*) encoding a basic region leucine zipper 60 protein. *bZIP60* was reported as a key transcription factor (TF) gene which is involved in the unfolded protein response (UPR; Iwata and Koizumi, 2005). UPR is induced, when unfolded or misfolded proteins are accumulated in the endoplasmic reticulum (ER), to refold or degrade the corresponding proteins (Walter and Ron, 2011). In plants, three bZIP-type proteins, bZIP17, bZIP28 as well as bZIP60, govern UPR in *Arabidopsis* (Iwata et al., 2008; Liu and Howell, 2010; Iwata and Koizumi, 2012; Howell, 2013). Those bZIP proteins reside in the ER under unstressed conditions, and once they sense ER stress, the former two proteins move to the Golgi apparatus and migrate to the nucleus after proteolytic processing by site 1 and site 2 proteases (S1P and S2P; Liu et al., 2007a,b). In contrast, *bZIP60* transcript is unconventionally spliced by an RNase activity of inositol-requiring enzyme 1 (IRE1) and the product of the resulting spliced *bZIP60* transcript is recruited to the nucleus. The *Arabidopsis* genome contains two *IRE1* genes, *AtIRE1A* (At2g17520) and *AtIRE1B* (At5g24360; Liu and Howell, 2010; Deng et al., 2011; Nagashima et al., 2011; Iwata and Koizumi, 2012; Srivastava et al., 2013). Several chemicals such as tunicamycin (TM) and dithiothreitol (DTT) are known to be UPR inducers. TM inhibits the *N*-linked glycosylation and DTT inhibits the disulfide bond formation, both of which are important for protein maturation.

Here, we aim to reveal whether Spm only acts on *bZIP60* or induces the whole UPR in *Arabidopsis*. We also address the upstream components in Spm-induced UPR pathways.

## Materials and Methods

### Plant Materials and Growth Condition

*Arabidopsis thaliana* ecotype Columbia-0 (Col-0) is used as wild type (WT). T-DNA insertion mutants, *ire1a* (SALK_018112, Deng et al., 2011, 2013), *ire1b* (SAIL_238_F07, Deng et al., 2011, 2013), *bzip60* (SALK_050203, Deng et al., 2011, 2013), *Atmpk6* (SALK_127507, Yoo et al., 2008; Zhou et al., 2009), *Atmpk3* (SALK_151594, Yoo et al., 2008; Zhou et al., 2009) were obtained from Dr. Stephen H. Howell (Iowa State University) and *Arabidopsis* Biological Resource Center, while *Atmkk9* (SAIL_60_H06, Xu et al., 2008) was kindly provided from Dr. Dongtao Ren (China Agricultural University). The double mutant, *ire1aire1b*, was obtained by crossing of *ire1a* and *ire1b*. Seeds were surface sterilized by 70% ethanol for 1min, then by 1% sodium hypochlorite solution containing 0.1% Tween-20 for 15 min, then followed by rinsing with sterilized water, three times. Sterilized seeds were sown on half strength MS medium (1% w/v agar, 1% w/v sucrose, 0.5 x MS salts, 0.05% vitamin B5, pH 5.6) and incubated in a growth chamber at 22°C under 16 h light/8 h dark conditions.

### Various Treatments

Ten-days-old *Arabidopsis* seedlings were incubated in half strength MS solution supplemented with or without Spm (0.5 mM) for 12 h under continuous light condition. PAs treatment: 0.5 mM concentration of PAs was used unless otherwise mentioned and incubated for 12 h. DTT treatment: DTT was applied at 2 mM concentration for 5 h in half strength MS solution. Lanthanum chloride (LaCl_3_) treatment: 10-days-old seedlings were incubated with or without La^3+^ (0.5 mM) for 12 h.

### Genome DNA-Polymerase Chain Reaction (PCR) and Reverse Transcription (RT)-PCR Analyses

Plant genome DNA was prepared by the procedure described by Murray and Thompson (1980). Total RNA was extracted from the respective plants samples using Sepasol-RNA I Super (Nacalai Tesque, Kyoto, Japan). Total RNA was treated with DNaseI (Takara DNase, Japan). First strand cDNA was synthesized from the DNaseI-treated RNA with ReverTra Ace (Toyobo Co. Ltd., Osaka, Japan), oligo-dT primer and dNTPs. *AtActin* was amplified using a specific primer pair (Supplementary Data Sheet S1) and used as a loading control. The amplified DNA fragments were separated by agarose-gels and visualized by ethidium bromide-staining.

### Quantitative Real-Time RT-PCR

Quantitative RT-PCR (qRT-PCR) analysis was performed by StepOne Real-Time PCR System (Applied Biosystems) using SYBR^®^ Green RT-PCR Kit (FastStart Universal SYBR Green Master, ROX). A standard curve was constructed from different genes and the values were normalized to *Actin* levels. The primers used for qRT-PCR were described in Supplementary Data Sheet S1.

### *bZIP60* Splicing Assay

*bZIP60* splicing assay was performed using the following primers; for detecting a unspliced form (*SPU*) and a spliced form (*SPS*), bZIP60-F primer and bZIP60-UB1 reverse primer and bZIP60-F primer and bZIP60-SB2 reverse primer, respectively, were used (Supplementary Data Sheet S1).

### Construction of Green Fluorescent Protein (GFP) Fusion Plasmids, Biolistic Bombardments, and Microscopic Observation

The basal GFP vector was constructed as described previously (Ono et al., 2012). Briefly, a GFP coding fragment was amplified by PCR using pGFP2 (provided by Dr. N.-H. Chua). The fragment was double-digested with *Bam*HI and *Sma*I, and subcloned into the pBI221 vector (Invitrogen), yielding pBI221GFP. Next, the internal two *Sac*I sites of *AtbZIP17* coding region were mutated without changing the amino acid sequence by two-step PCR. Then, a second PCR was performed on the respective mixtures of the first PCR products using the primers, AtbZIP17-SmaI-F and AtbZIP17-SacI-Rv (Supplementary Data Sheet S1). The coding region of *AtbZIP60* was amplified by PCR. The resulting *AtbZIP17* and *AtbZIP60* fragments digested with *Sma*I and *Sac*I were subcloned into the corresponding restriction enzyme sites of pBI221GFP, yielding pBI221GFP-AtbZIP17 and pBI221GFP-AtbZIP60, respectively. The resulting GFP-AtbZIPs’ constructs were bombarded into onion bulbs by particle bombardment. ER-targeting DsRED plasmid (Okamoto et al., 2008) or nuclear-targeting mCherry-VirD2_NLS plasmid (CD3-1106, purchased from ABRC) was co-bombarded as the ER- and nuclear-markers. After incubating the onion bulbs with or without Spm (or DTT) at 22°C for 16 h under darkness, onion epidermal cells were peeled and placed onto glass slides and observed with a fluorescence microscope (BX61; Olympus).

### *Arabidopsis* Transgenic Plants Overexpressing *SPMS*

Three independent lines of the transgenic *Arabidopsis* overexpressing *SPMS* were used. The detailed procedure to generate the transgenics was described in Sagor et al. (2012a).

### PA Analysis

Polyamine analysis was performed as described in Naka et al. (2010). In brief, plant samples (0.3–0.5 g per sample) were pulverized with a mortar and pestle under liquid nitrogen. Five volumes (2.5 mL per 0.5 g of plant sample) of 5% (v/v) cold perchloric acid were added to the resulting fine powders. The mixtures were transferred to plastic tubes and kept on ice for 1 h. After centrifugation at 15,000 × *g* for 30 min at 4°C, the supernatants were combined and filtered using a filter syringe (pore size, 0.2 μm). One milliliter of 2 N NaOH was added to 1.5 mL of plant extract and mixed thoroughly. Then 10 μL of benzoyl chloride was added and the mixture was incubated at room temperature for 20 min. After adding 2 mL of saturated NaCl and 2 mL of diethyl ester, samples were vigorously mixed and then centrifuged at 3,000 × *g* for 10 min at 4°C for phase-separation. An aliquot (1.5 mL) of the organic solvent phase was evaporated and the residue was resuspended in 50 μL of methanol. Benzoylated PAs were analyzed with a programmable Agilent 1200 liquid chromatograph using a reverse-phase column (4.6 mm × 250 mm, TSK-GEL ODS-80Ts, TOSOH, Tokyo, Japan) and detected at 254 nm. One cycle of the run took 60 min in total with a flow rate of 1 mL/min at 30°C, i.e., 42% acetonitrile for 25 min for PA separation, increased to 100% acetonitrile over 3 min, 100% acetonitrile for 20 min for washing, decreased to 42% acetonitrile over 3 min, and finally 42% acetonitrile for 9 min.

### Statistical Analysis

Student’s *t*-tests were used for statistical analysis and were performed using Microsoft Excel statistical tools.

## Results

### Exogenously Applied Spm Induces *bZIP17* and *bZIP28* as Well as *bZIP60* and their Target Genes

**FIGURE 1:**
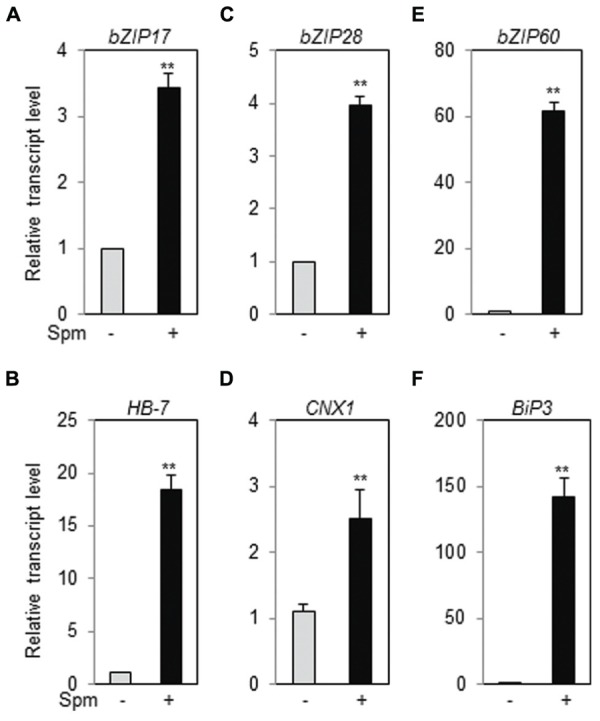
**Exogenous Spm induces the expression of *bZIP17*, *bZIP28*, and *bZIP60* and their target genes**. qRT-PCR was performed using the cDNAs prepared from control- and Spm-treated *Arabidopsis* seedlings. **(A)** *bZIP17*; **(B)** *HB-7*; **(C)** *bZIP28*; **(D)** *CNX1*; **(E)** *bZIP60*; **(F)** *Bip3*. The transcript level in control *Arabidopsis* plant was set as 1 and relatively displayed. Values are mean (+SD) from three independent experiments. Asterisks indicate significant difference (^∗∗^*P* < 0.01).

Ten-days-old *Arabidopsis* ecotype (Col-0) seedlings were treated with Spm or DTT (as positive treatment for UPR induction) and qRT-PCR was performed. Spm induced *bZIP17*, *bZIP28* and *bZIP60*, and their respective target genes, *HB-7*, *CNX1*, and *BiP3*, respectively, as similar as DTT did (**Figure 1**, Supplementary Figure S1). The result clearly indicates that Spm is a novel UPR inducer.

### Spm Activates the IRE1-Mediated *bZIP60* Splicing

**FIGURE 2:**
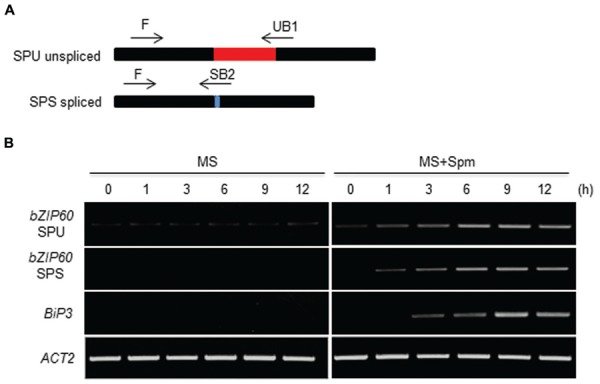
**Time-course analysis of *bZIP60* splicing upon Spm treatment. (A)** A schematic representation of *bZIP60* transcript with the position and orientation of primer pairs used for detecting the unspliced (*SPU*)- and spliced (*SPS*)-forms of *bZIP60*. **(B)** Time course RT-PCR analysis of *SPU* and *SPS* forms of *bZIP60* transcript and its downstream target *BiP3* transcript at indicated time intervals in control and Spm-treated seedlings. *Actin* was used as an internal control.

**FIGURE 3:**
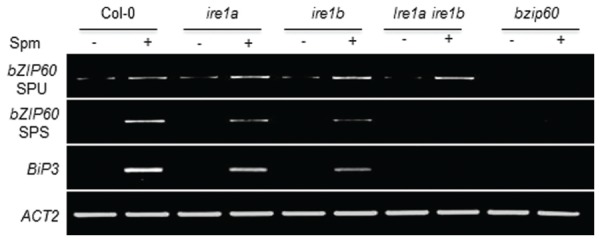
**Spm-induced *bZIP60* splicing occurs in an IRE1-dependent manner**. Ten-days-old seedlings of Col-0 (control), *ire1a* (SALK_018112, Deng et al., 2011, 2013; Nagashima et al., 2011) mutant, *ire1b* (SAIL_238_F07) mutant, and *ire1aire1b* double mutant, and *bzip60* (SALK_050203) mutant were treated with half strength MS solution as a control (-) and half strength MS solution containing 0.5 mM Spm (+). Results of RT-PCR for expression analysis of *SPU* and *SPS* forms of *bZIP60* and its downstream gene *BiP3* were displayed. *Actin* was used as an internal control.

Canonical UPR inducers activate *bZIP60* splicing, which is mediated by IRE1 (Deng et al., 2011, 2013). Thus we addressed this point. At 1 h after Spm treatment, the unspliced form (*SPU*) of *bZIP60* transcripts was accumulated. At a little time gap after the SPU detection, the spliced form (*SPS*) of *bZIP60* transcripts was detected, which followed by *BiP3* induction (**Figure 2**). To further prove the IRE1 involvement in the process, we used *ire1a, ire1b* and *ire1a ire1b* (see Supplementary Figure S2) double mutant along with *bzip60* mutant (Deng et al., 2011, 2013). In *ire1a* mutant, the levels of the *SPS* form of *bZIP60* transcript and *BiP3* transcript were slightly lowered compared to those of WT after Spm treatment. In *ire1b* and *ire1a ire1b* mutants, the transcriptional attenuation of the *SPS-bZIP60* and *BiP3* was strikingly observed (**Figure 3**). It shows that IRE1B is mainly participated in Spm-induced *bZIP60* unconventional splicing.

### Endogenous Spm Induces *bZIP17, bZIP28*, and bZIP60 and their Target Genes

**FIGURE 4:**
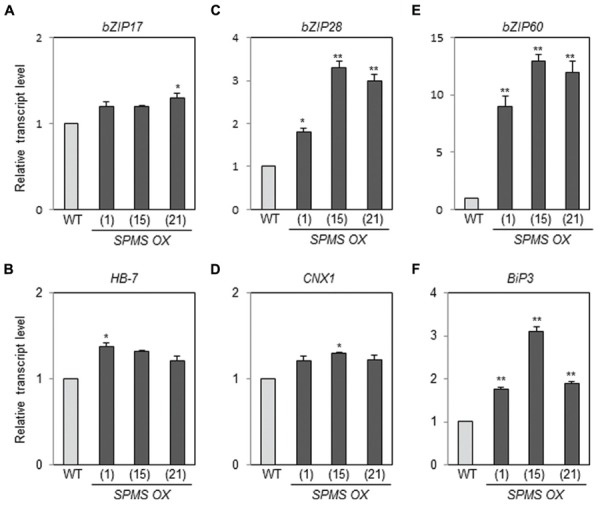
**Endogenous Spm induces the expression of *bZIP17*, *bZIP28*, and *bZIP60* and their target genes.** The levels of *bZIP17* **(A)**, *bZIP28* **(C)**, and *bZIP60* **(E)** transcripts, and their respective target gene transcripts [*HB-7* **(B)***, CNX1* **(D)**, and *BiP3* **(F)**] in the *SPMS_OX* transgenic lines were quantitatively determined by qRT-PCR analysis using the primer pairs listed in Supplementary Data Sheet S1. Values are mean (+SD) from three independent experiments. Asterisks indicate significant difference (^∗^*P* < 0.05 and ^∗∗^*P* < 0.01).

We have generated the transgenic *Arabidopsis* overexpressing *SPMS* (Sagor et al., 2012a). The three independent lines, designated as *SPMS OX*_1, *SPMS OX*_15, and *SPMS OX*_21, were used in this study. In those transgenics, the *SPMS* transcripts were remarkably accumulated (Supplementary Figure S3A) and concomitantly Spm content increased significantly, in contrast, Spd content decreased (Supplementary Figure S3B). We examined the transcript levels of *bZIP17*, *bZIP28*, and *bZIP60* in the Spm-enriched *Arabidopsis* plants. The levels of *bZIP17*, *bZIP28*, and *bZIP60* transcripts became approximately 1.2∼1.3-fold, 2- to 3-fold, and 9- to 12-fold, respectively, in the *SPMS OX* transgenics compared those in WT (**Figures 4A,C,E**). The levels of *BiP3* transcripts became clearly higher than those in WT (**Figure 4F**), whereas those of *HB-7* and *CNX1* were still higher than those in WT but not so much significant (**Figures 4B,D**). In the Spm-enriched plants, homeostatic regulation to suppress the constitutive UPR may be operated. Anyway, not only exogenously applied Spm but also high endogenous Spm are able to induce the UPR. PA specificity assay showed that T-Spm has a similar inducing activity as Spm does, and Spd has a weaker activity but Put has no such activity (Sagor et al., 2012b; Supplementary Figure S4, data not shown). Spm and T-Spm induced the expression of *bZIP60* even at 100 μM (Supplementary Figure S4).

### Spm Recruits *bZIP17* and *bZIP60* from ER to Nucleus

**FIGURE 5:**
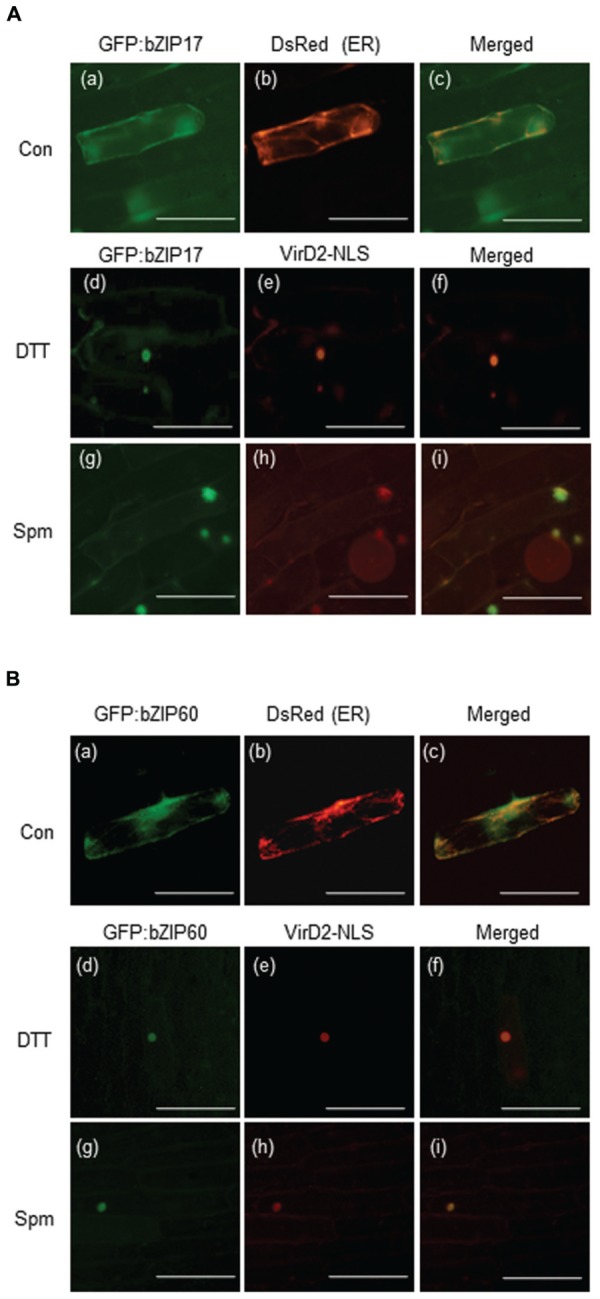
**Recruitment of bZIP17 and bZIP60 proteins from ER to nucleus upon Spm treatment. (A)** GFP:bZIP17 fusion protein under normal condition (a–c); DTT treatment (d–f); and Spm treatment (g–i). **(B)** GFP:bZIP60 fusion protein under normal condition (a–c); DTT treatment (d–f); and Spm treatment (g–i). The plasmids encoding GFP:bZIP17 and GFP:bZIP60 proteins were co-bombarded to onion bulbs with the plasmid encoding an ER marker peptide tagged with Ds-Red or with the plasmid encoding a nuclear marker protein VirD2_NLS using a particle gun. The bombarded samples were treated with or without Spm for 16 h under darkness at 22°C. The samples were also treated with DTT as a positive reference. Then onion epidermal cell layers were peeled off and observed by a fluorescence microscope (BX61; Olympus). Bar indicates 10 μm.

*bZIP17*, *bZIP28*, and *bZIP60* encode the bZIP-type TF proteins. To accomplish their roles as TFs, their products have to reach to nuclei. Here we generated the GFP:bZIP17 and GFP:bZIP60 fusion plasmids and delivered them to onion epidermal cells. Before Spm treatment, both the fusion proteins seemed to locate in ER because the green fluorescent signals overlapped with the red fluorescent signals emitted by the ER-positive marker (**Figures 5Aa–c,Ba–c**). Upon Spm treatment, the green fluorescent signals from both the fusion proteins were merged to the red fluorescent signals emitted from the nucleus-targeting marker plasmid, indicating that bZIP17 and bZIP60 proteins were recruited to nucleus after Spm treatment (**Figures 5Ag–i,Bg–i**) as well as after DTT treatment (**Figures 5Ad–f,Bd–f**).

### Ca^2+^-Influx is One of the Upstream Components of Spm-Induced UPR

**FIGURE 6:**
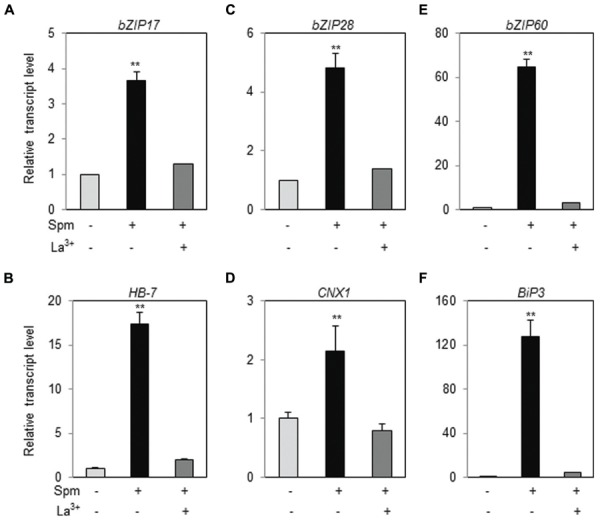
**Ca^2+^-channel blocker alleviates the UPR induction by Spm**. qRT-PCR was performed using the cDNAs prepared from control-, Spm-treated, and Spm and La^3+^-co-treated *Arabidopsis* seedlings. **(A)** *bZIP17*; **(B)** *HB-7*; **(C)** *bZIP28*; **(D)** *CNX1*; **(E)** *bZIP60*; **(F)** *Bip3*. The transcript level in control *Arabidopsis* plant was set as 1 and relatively displayed. Values are mean (+SD) from three independent experiments. Asterisks indicate significant difference (^∗∗^*P* < 0.01).

In tobacco plant, we have demonstrated that Ca^2+^-influx to cytoplasm and the activation of the mitogen-activated protein kinase (MAPK) cascade consisting of NtMEK2-SIPK/WIPK are prerequisite for triggering a Spm-signaling pathway (Takahashi et al., 2003, 2004). First, therefore, we addressed whether Ca^2+^-influx to cytoplasm is required for UPR induction by Spm treatment. We applied a Ca^2+^-channel blocker, La^3+^, to the plant samples when Spm was applied. Upregulation of *bZIP17*, *bZIP28*, and *bZIP60* was totally alleviated in the plants co-treated with Spm and La^3+^ (**Figures 6A,C,E**). Induction of the target genes, *HB-7*, *CNX1* and *BiP3*, of bZIP17, bZIP28, and bZIP60 was also blocked by La^3+^ treatment (**Figures 6B,D,F**). The results indicate that Spm-induced UPR pathway requires the enhanced Ca^2+^ influx to cytoplasm.

### A MAPK Cascade is Another Upstream Component of Spm-Induced UPR

**FIGURE 7:**
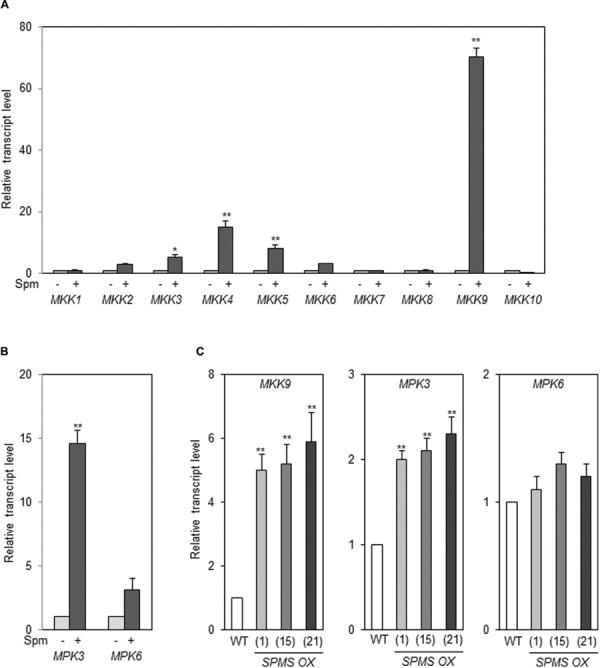
**Spm upregulates *MKK9, MPK3*, and *MPK6*. (A)** Exogenous Spm upregulated *MKK9* among 10 *MKK*s. **(B)** Exogenous Spm upregulated *MPK3* and *MPK6*. **(C)** The transcript levels of *MKK9*, *MPK3*, and *MPK6* in the *SPMS_OX* transgenic lines. The levels of *MKK9*, *MPK3*, and *MPK6* transcripts were quantitatively determined by qRT-PCR using the primer pairs listed in Supplementary Data Sheet S1. Values are mean (+SD) from three independent experiments. Asterisks indicate significant difference (^∗^*P* < 0.05 and ^∗∗^*P* < 0.01).

In *A. thaliana*, there are more than 60 MAPK kinase kinases (MAP3K), 10 MAPKKs (MKK), and 20 MAPKs (MAPK Group, 2002). Therefore, we examined whether any *MKK*s are up-regulated by exogenously applied Spm. Of 10 *MKK*s, *MKK9* was strikingly induced by Spm (**Figure 7A**). The levels of *MKK4* and *MKK5* transcripts were also accumulated at the lesser extent compared to those of *MKK9* (**Figure 7A**). The previous research showed that the downstream MAPKs of MKK9 are MPK6 (SIPK ortholog) and/or MPK3 (WIPK ortholog; see review Colcombet and Hirt, 2008; Yoo et al., 2008; Zhou et al., 2009; Rodriguez et al., 2010). In addition to *MKK9*, *MPK3* was identified as Spm-responsive gene in a SuperSAGE analysis (Mitsuya et al., 2009). So we tested the expressional response of *MPK3* and *MPK6* upon Spm treatment. The qRT-PCR result showed that expression of both *MPK3* and *MPK6* was up-regulated by Spm treatment (**Figure 7B**). Furthermore, we quantified the transcript levels of *MKK9*, *MPK3*, and *MPK6* in the Spm-enriched transgenic plants, and found that *MKK9* and *MPK3* transcripts were distinctly accumulated and *MPK6* transcripts increased to approximately 1.2–1.4-fold levels in the *SPMS OX* plants compared to those in WT (**Figure 7C**).

### Spm-Induction of UPR Pathway is Mediated by the MKK9-MPK3/MPK6 Cascade

**FIGURE 8:**
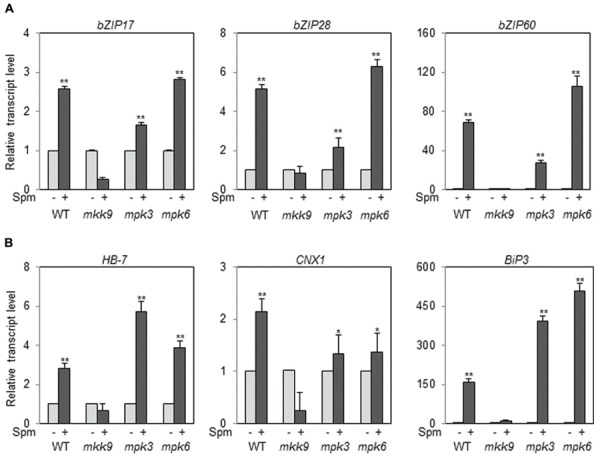
**The expression of *bZIP17*, *bZIP28*, and *bZIP60***(A)** and their target genes **(B)** in the loss-of-function mutants, *mkk9*, *mpk3*, and *mpk6*, upon Spm treatment**. The expression levels of the three bZIP genes and their target genes in *mkk9*, *mpk3*, and *mpk6* were determined by qRT-PCR. The experiments were performed using three independent biological samples. The mean + SD were displayed. Asterisks indicate significant difference (^∗^*P* < 0.05 and ^∗∗^*P* < 0.01).

Then the question arose whether all the paths of bZIP17, bZIP28, and bZIP60 in the UPR are positioned downstream of the MKK9-MPK3/MPK6 cascade. To answer the issue, we used the T-DNA insertion mutants of *MKK9*, *MPK3*, and *MPK6*. The T-DNA insertion sites in *MKK9*, *MPK3*, and *MPK6* were displayed in Supplementary Figure S5A. The homogeneity of the T-DNA insertion in the mutants, *mkk9*, *mpk3*, and *mpk6*, was confirmed by the genome DNA-PCR (Supplementary Figures 5B–D; Xu et al., 2008; Yoo et al., 2008; Zhou et al., 2009). The levels of *MKK9*-, *MPK3*-, and *MPK6*-transcripts in the *mkk9*, *mpk3*, and *mpk6* mutants were almost null in relative to those of WT, respectively (Supplementary Figures 5E–G). Using the loss-of-function mutants, we examined the levels of *bZIP17*, *bZIP28*, and *bZIP60* transcripts upon Spm treatment. The expression of *bZIP17*, *bZIP28*, and *bZIP60* was not enhanced by Spm treatment in the *mkk9* mutant (**Figure 8A**). The expression of their target genes was also not induced by Spm in the *mkk9* mutant (**Figure 8B**). The expression of the bZIP genes and their target genes was more or less similarly induced by Spm in the *mpk3* and *mpk6* mutants. The latter result may be explained that MPK3 and MPK6 have redundant function to transduce the Spm-signal. It should be noted that Spm-signaling pathway was blocked in *Nicotiana benthamiana* in which both, *SIPK* and *WIPK*, were simultaneously silenced but neither in *SIPK*-silencing nor in *WIPK*-silencing *N. benthamiana* (Takahashi et al., 2004). Collectively it indicates that the induction of *bZIP17*, *bZIP28*, and *bZIP60* depends on the MKK9-MPK3/MPK6 cascade.

## Discussion

### Spm Activates Three Pivotal bZIP Genes and their Target Genes in UPR

Previous research showed that exogenous Spm induces the expression of *Arabidopsis bZIP60* and its ortholog, *NtbZIP60*, of *N. tabacum* (Tateda et al., 2008; Mitsuya et al., 2009). We addressed whether Spm induces *bZIP60* specifically or rather induces the whole UPR process. Here, we showed that Spm induces not only *bZIP60* but also *bZIP17* and *bZIP28* expression (**Figures 1** and **4**). In the case of *bZIP60*, an IRE1-dependent *bZIP60* splicing process was also activated by Spm (**Figures 2** and **3**). We speculate that the proteolytic bZIP17 and bZIP28 processing by S1P and S2P is activated by Spm because their downstream target genes such as *HB-7*, *RD20*, *CNX1*, and *CRT2* were concomitantly upregulated (**Figure 1**, data not shown). In addition, in Spm-enriched *Arabidopsis* plants, the three *bZIP* genes were induced (**Figure 4**). We further showed that Spm can recruit bZIP17 and bZIP60 proteins to nuclei in plant cells (**Figure 5**). Taken together, we conclude that Spm is a novel inducer of UPR.

### Spm-Induced UPR Pathway is Mediated by the MKK9-MPK3/MPK6 Cascade

Previous work showed that Spm activates two kinds of MAPKs, SIPK and WIPK, and this activation requires the production of reactive oxygen species and Ca^2+^ influx in tobacco leaves (Takahashi et al., 2003, 2004). Our result showed that MKK9-MPK3/MPK6 is the MAPK cascade for Spm-induced UPR in *Arabidopsis*. The *Arabidopsis* MAPK signaling pathways are involved in various biological processes (Colcombet and Hirt, 2008; Rodriguez et al., 2010). The MKK9-MPK3/MPK6 cascade was used in ethylene signaling (Yoo et al., 2008) and was also involved in the phytoalexin production, and in salt stress response in *Arabidopsis* (Xu et al., 2008). Furthermore, our result showed that, in *mkk9* mutant, Spm-induced *bZIP17*, *bZIP28*, and *bZIP60* expression was significantly alleviated (**Figure 8**). Moreover, the Spm-induced expression of the respective target genes, *HB-7*, *CNX1*, and *BiP3*, was also abrogated in the *mkk9* mutant (**Figure 8**). The result suggests that Spm-evoked Ca^2+^-elevation activates the MKK9-MPK3/MPK6 cascade, which triggers the *bZIP17*, *bZIP28*, and *bZIP60* induction. It should be noted that, in mammalian system, IRE1 activates JNK protein kinases (Urano et al., 2000). To know whether *Arabidopsis* IRE1 can activate the MKK9-MPK3/MPK6 pathway is of interest.

### How does Spm Induce UPR?

We noticed that the Spm-induced expression profile of three UPR key bZIP genes and their target genes differs from the one induced by the typical UPR inducers, DTT and TM. Martinez and Chrispeels (2003) described that the respective UPR agents have different effects; i.e., the genes induced by TM and DTT were different in *Arabidopsis*. It is quite reasonable because TM affects *N*-glycosylation whereas DTT changes the cellular redox state (Liu and Howell, 2010). Then the question arose how Spm induces the UPR. Spm-induced expression of *bZIP17, bZIP28*, and *bZIP60* genes was blocked by a Ca^2+^ channel blocker (**Figure 6**). Spm- and T-Spm-deficient *Arabidopsis* plants cannot grow well in Ca^2+^-depleted MS medium (Yamaguchi et al., 2006), suggesting that the tetraamines are involved in Ca^2+^ dynamics and homeostasis. PAs are known to modulate Ca^2+^ dynamics via control of cation channel activity (Dobrovinskaya et al., 2000; Pottosin and Shabala, 2014). Furthermore, the action potential to regulate cation channels is the order of Spm > Spd ≥ Put, which may explain the PA specificity to evoke UPR induction. As exogenously applied T-Spm also induces Spm-responsive genes at the higher degree compared to Spm (Sagor et al., 2012b), T-Spm may possess a similar or the higher action potential in terms of modulating cation channels in relative to Spm. It is known that thapsigargin, another UPR inducer, affects Ca^2+^ homeostatic balance. Thapsigargin leads to ER Ca^2+^ depletion due to inhibition of the Sarco/ER Ca^2+^ ATPase (SERCA; Xu et al., 2008). Taken together, we hypothesize that Spm induces UPR pathway through affecting Ca^2+^ dynamics. Of course further study is needed to substantiate the hypothesis.

### Physiological Relevance of Spm-Induced UPR

It was demonstrated that the polyamine Spm, exogenously applied or endogenously enriched, has a protective role in the defense response of plants against both abiotic and biotic stresses like salt, drought and heat stresses, and attack by viral pathogens (Yamaguchi et al., 2006, 2007; Mitsuya et al., 2009; Sagor et al., 2012a; Tiburcio et al., 2014; Berberich et al., 2015). A protective role was also shown for components of the UPR. When the *bZIP60* ortholog of *N. benthamiana* was silenced by a virus-induced gene silencing approach, the host plant became hypersensitive to non-host bacterial pathogen (Tateda et al., 2008). *bZIP60* was upregulated when the avirulent CMV-Y strain, but not the virulent CMV-B2 strain, was used to infect *Arabidopsis* ecotype C24 which carries the resistance gene, *RCY1*, to CMV-Y (Takahashi et al., 1994; Mitsuya et al., 2009). In parallel Spm synthase gene was upregulated and Spm content increased in the C24-CMV-Y pathosystem (Mitsuya et al., 2009). We assume a link inasmuch as the accumulated Spm activates UPR. The protection against viral infection could be accomplished indirectly by degradation of the excess Spm by polyamine oxidases which produce H_2_O_2_. H_2_O_2_ would then be transmitted to the surrounding tissue of the infection site, thereby inducing the UPR in such cells. Since the virus is unable to replicate properly while UPR takes place the tissue is protected. A distinct role of UPR in plant immunity mediated by IRE1/bZIP60 has been shown by Moreno et al. (2012). Those authors demonstrated that SA, an important phytohormone in immune response, can induce IRE1/bZIP60-mediated UPR. Nagashima et al. (2014) further showed that SA activates two signaling arms, namely bZIP28 and bZIP60, of the UPR in *Arabidopsis*. Those results suggest that bZIP60 (or its ortholog) has a defensive role against avirulent pathogens. In contrast, bZIP17 and bZIP28 were found to be rather associated with abiotic stresses such as drought, ABA and high salinity, and heat stress, respectively (Soderman et al., 1996; Liu et al., 2007b; Liu and Howell, 2010; Howell, 2013), for which a defensive role of Spm has also been demonstrated as mentioned above. In conclusion, the broad effect of Spm in the stress response of plants could originate, at least partly, in the capacity of Spm as an UPR inducer.

## Conflict of Interest Statement

The Guest Associate Editor Taku Takahashi declares that, despite having previously collaborated with the authors Masaru Niitsu and Tomonobu Kusano, the review process was handled objectively. The authors declare that the research was conducted in the absence of any commercial or financial relationships that could be construed as a potential conflict of interest.
