# Supplementary material for: The polyamine spermine induces the unfolded protein response via the MAPK cascade in Arabidopsis
Source: Front Plant Sci. 2015 Sep 10;6:687. doi: 10.3389/fpls.2015.00687 (PMC4565113; doi:10.3389/fpls.2015.00687)
Supplement: Supplementary file 1 [file Data_Sheet_1.DOCX]

***Supplementary Material***

**The polyamine spermine induces the unfolded protein response via the MAPK cascade in Arabidopsis**

**G.H.M. Sagor^1,†^, Pratima Chawla^1,†^, Dong Wook Kim^1,#^, Thomas Berberich^2^, Seiji Kojima^1,3^, Masaru Niitsu^4^, Tomonobu Kusano^1*^**

*** Correspondence:** Tomonobu Kusano: kusano@ige.tohoku.ac.jp

1. **Supplementary Data**

There are 4 supplemental Figures and 1 supplemental table.

1. **Supplementary Figures and Tables**

## Supplementary Figures

**Supplementary Figure 1.**

**Exogenous Spm induces the expression of *bZIP17*, *bZIP28* and *bZIP60* and their target genes as similar as DTT does.** RT-PCR was performed using the cDNAs prepared from Spm- and DTT-treated Arabidopsis seedlings. *bZIP17*, *HB-7*, *bZIP28*, *CNX1*, *bZIP60* and *Bip3* were examined. *ACT2* was used as a loading control.

**Supplementary Figure 2.**

**Validation of *ire1a, ire1b* and *ire1aire1b* mutants. A,** Schematic representation of genome organization and T-DNA insertion sites of *ire1a* and *ire1b* mutants. Dark and light grey boxes indicate coding regions in *ire1a* and *ire1b,* respectively and white boxes represent untranslated regions. **B,** RT-PCR analysis of WT (Col-0), *ire1a*, *ire1b* and *ire1a ire1b* mutants by using the specific primers mentioned in Table S1.

**Supplementary Figure 3.**

**Generation of the transgenic Arabidopsis plants overexpressing *SPMS*.** **A**, The *SPMS* transcript levels in the three *SPMS_OX* transgenic lines. **B**, PA contents in the three *SPMS_OX* transgenic lines. The experiments were performed using three biological samples. The means + SD were calculated. Asterisks indicate significant difference (^**^*P* < 0.01).

**Supplemental Figure 4.**

This figure was cited from Sagor G. H. M. (2012) Differential roles of tetraamines, spermine and thermospermine, in *Arabidopsis thaliana*. Saito Ho-on Kai Mus. Nat. Hist. Res. Bull., No. 76, March, pp. 44.

**
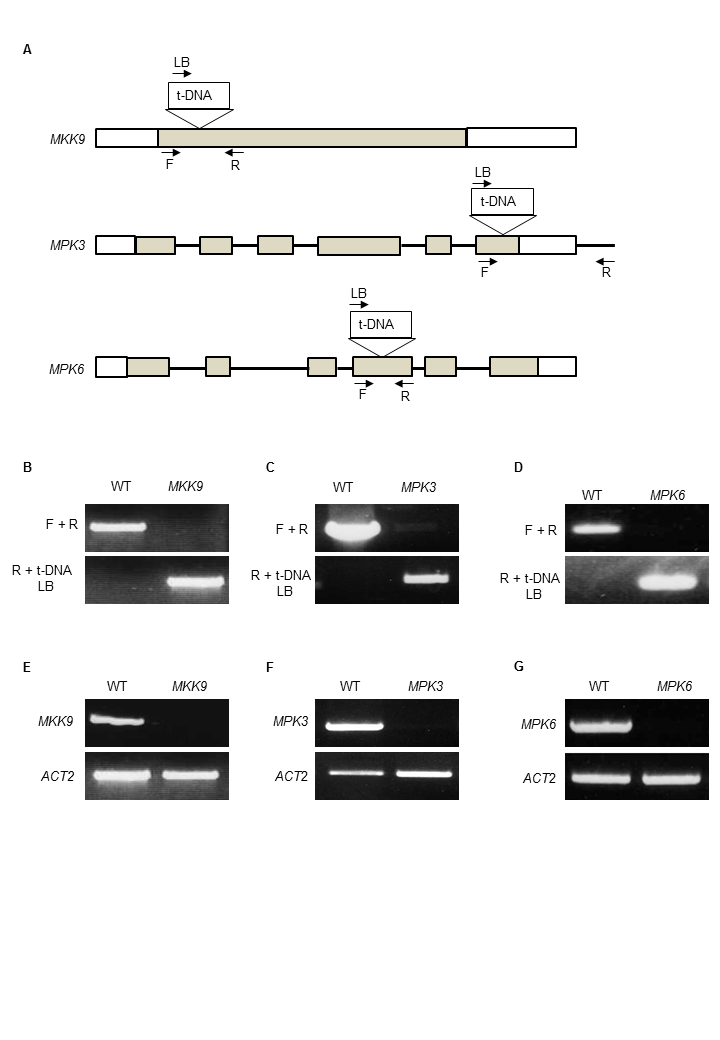
**

**Supplementary Figure 5.**

**Confirmation of the loss-of-function mutants, *mkk9*, *mpk3* and *mpk6*. A**, Schematic illustration of the genome organization of *MKK9*, *MPK3* and *MPK6* and the T-DNA insertion sites in their genes. The primer position and their orientation were also illustrated by arrows. Genomic PCR analysis of (**B**) *MKK9*; (C) *MPK3*; (D) *MPK6* genes. Expression analysis of (**E**) *MKK9*; (**F**) *MPK3*; (**G**) *MPK6* genes. *ACTIN* was used as a loading control.

## Supplementary Tables

**Supplementary Table 1.** Primers used in this study.
